# *Clostridium perfringens*-Induced Host-Pathogen Transcriptional Changes in the Small Intestine of Broiler Chickens

**DOI:** 10.3390/pathogens10121607

**Published:** 2021-12-10

**Authors:** Mingmin Lu, Baohong Yuan, Xianghe Yan, Zhifeng Sun, Hyun S. Lillehoj, Youngsub Lee, Calder Baldwin-Bott, Charles Li

**Affiliations:** 1 Animal Biosciences and Biotechnology Laboratory, Beltsville Agricultural Research Center, Agricultural Research Service-US Department of Agriculture, Beltsville, MD 20705, USA; Mingmin.lu@usda.gov (M.L.); Yuanbaohong@gdpu.edu.cn (B.Y.); Zhifeng.sun@usda.gov (Z.S.); Hyun.lillehoj@usda.gov (H.S.L.); Youngsub.Lee@usda.gov (Y.L.); Calderbaldwinbott@gmail.com (C.B.-B.); 2 School of Basic Medicine Sciences, Guangdong Pharmaceutical University, Guangzhou 510006, China; 3 Environmental Microbial and Food Safety Laboratory, Beltsville Agricultural Research Center, Agricultural Research Service-US Department of Agriculture, Beltsville, MD 20705, USA; 4 Eleanor Roosevelt High School, Greenbelt, MD 20770, USA

**Keywords:** *C. perfringens*, necrotic enteritis, RNA-sequencing, broiler chickens, host-pathogen interactions

## Abstract

*Clostridium perfringens* is an important opportunistic pathogen that may result in toxin-mediated diseases involving food poisoning/tissue gangrene in humans and various enterotoxaemia in animal species. It is a main etiological agent for necrotic enteritis (NE), the most financially devastating bacterial disease in broiler chickens, especially if raised under antibiotic-free conditions. Importantly, NE is responsible for losses of six billion US dollars annually in the global poultry industry. To investigate the molecular mechanisms of *C. perfringens*-induced pathogenesis in the gut and its microbiome mRNA levels in *C. perfringens*-infected and non-infected hosts, we used RNA sequencing technology to perform transcriptional analysis of both host intestine and microbiome using our NE model. The growth rate was significantly impaired in chickens infected by *C. perfringens.* In total, 13,473 annotated chicken genes were differentially expressed between these two groups, with ninety-six genes showing statistical significance (|absolute fold changes| > 2.0, adjusted *p* value < 0.05). Genes involved in energy production, MHC Class I antigen, translation, ribosomal structures, and amino acid, nucleotide and carbohydrate metabolism from infected gut tissues were significantly down-regulated. The upregulated genes were mainly engaged in innate and adaptive immunity, cellular processes, genetic information processing, and organismal systems. Additionally, the transcriptional levels of four crucial foodborne pathogens were significantly elevated in a synergic relationship with pathogenic *C. perfringens* infection. This study presents the profiling data that would likely be a relevant reference for NE pathogenesis and may provide new insights into the mechanism of host-pathogen interaction in *C. perfringens*-induced NE infection in broiler chickens.

## 1. Introduction

*Clostridium perfringens* is a Gram-positive, spore-forming anaerobe bacterium inducing opportunistic infection in humans and livestock worldwide [1]. *C. perfringens* induced-necrotic enteritis (NE) results in histotoxic illnesses with various clinical symptoms ranging in severity from diarrhea to enteritis or enterotoxaemia [2]. The successful transmission and colonization of *C. perfringens* strains in the hosts are mainly attributable to their survival patterns including oxygen sensitivity, sporulation, germination, and rapid proliferation capacity [3]. An arsenal of toxins (>20 identified toxins) produced by *C. perfringens* accounts for its virulence and pathogenesis in the host intestine or other organs [4]. On the basis of toxinotyping classification, namely based on the production of enterotoxin (CPE), NetB, α-, β-, ε-, and ι- toxin, *C. perfringens* isolates are categorized into seven types (A-G) [5]. In the United States, *C. perfringens* is one of the primary causes of non-foodborne diarrheal and foodborne diseases, with annual infected cases of approximately one million. The most common vehicle for *C. perfringens* infections is poultry and undercooked meat [6,7]. Epidemiological surveillance data of the CDC indicated that *C. perfringens* is responsible for 4% of hospitalizations, 10% of illnesses, and 5% of outbreaks for food poisoning diseases [8].

Regardless of the outbreaks in human hosts, the widespread occurrence of *C. perfringens* infections in domestic animals, including equine, swine, bovine, and poultry, has caused substantial global economic losses and has been regarded as a notable constraint on the livestock industry [3]. In poultry, *C. perfringens*-induced NE has been unequivocally recognized as one of the most leading intestinal diseases in turkeys and chickens, characterized by decreased growth rate and elevated feed conversion subclinically, and diagnosed with gaseous lesions and mucosa necrosis of the small intestine at post-mortem [9,10]. There is an indication that the onset of poultry NE is associated with an array of virulence factors, including pore-forming toxins like α-toxin, TpeL, NetB and hydrolytic enzymes like collagenase [11]. In most instances, improper feeding and management practices like high stocking density and high-protein diets may interrupt gut immune homeostasis and promote *C. perfringens* proliferation and NE infection [12,13]. In addition, coccidial infections have been the predisposing factor for poultry NE by facilitating *C. perfringens* multiplication that damages the intestinal epithelial layer [13,14,15].

The emergence of antimicrobial resistance in *C. perfringens* isolates from the field poses a significant threat to public health, given their capability to generate a variety of lethal toxins [3]. For sustainable poultry production, timely development of alternatives to antibiotic growth promoters (AGPs) is needed to counteract the increasing antimicrobial resistance [10]. A number of non-antimicrobial strategies, e.g., plant extracts, organic acids, essential oils, prebiotics, and probiotics, have been demonstrated to deliver encouraging outcomes for the control of NE in broiler chicks [16]. However, the protective efficacy of these therapeutics on the mitigation of NE is inconsistent and variable [17,18]. Therefore, the rational design of novel intervention against NE relies on understanding host defense mechanisms [3]. Preliminary studies revealed that host protective immunity against *C. perfringens* infection was associated with a cascade of effector mechanisms. The interactions of *C. perfringens* pathogen-associated molecular patterns with host Toll-like receptors may trigger the activation of immune cells like T helper (Th) cells and dendritic cells, thereby resulting in the secretion of cytokines like interleukin (IL)-10, IL-17, and interferon-γ [19,20,21,22]. However, the underlying molecules involved in modulating host immune responses at the host-NE interface remain largely unknown. Gut microbiota belongs to the host central defense system and modulates host responses to prevent pathogen colonization and infection [23]. Although changes in gut microbiota have been implicated in NE pathogenesis, the causal relationship between microbiota changes with NE development is still uncertain [24]. Hence, in this study, we aimed to investigate the effects of *C. perfringens* infection on the host small intestine at the molecular level and explore the impact of *C. perfringens* infection on the major foodborne microbiota members in the gut. With next-generation RNA-Seq sequencing technology, we identified a plethora of differentially expressed genes (DEGs) in the small intestinal mucosa in response to *C. perfringens* infection. Many fundamental molecular processes associated with NE pathogenesis were also deciphered. In addition, we revealed the specific changes of other foodborne pathogens from the gut microbiota profiling during *C. perfringens* infection. The profiling data presented here would be a relevant reference for the studies of *C. perfringens*-induced NE infection in broiler chickens and may enhance our understanding of host-pathogen interaction in NE pathogenesis.

## 2. Materials and methods

### 2.1. Animals Used for This Study

Ross 708 broiler chicks at the age of one day were obtained from Longenecker’s Hatchery (Elizabethtown, PA, USA). They were housed in Petersime starter brooder units and later in finisher units, and were supplied with feed and water *ad libitum*. The Institutional Animal Care and Use Committee of Beltsville Agricultural Research Center approved the animal studies (Animal use protocol Number 17-027, Approved in December 2017).

### 2.2. Challenge Infection by C. perfringens and RNA Extraction

The *C. perfringens* strain *netB*^+^/*tpeL*^+^ LLY_TpeL17 was used as the challenge inoculum to infect the broiler chickens as described elsewhere [25]. Briefly, the *netB*^+^/*tpeL*^+^ LLY_TpeL17 strains were propagated in CMG medium (Anaerobe Systems, Morgan Hill, CA, USA) and then in BYC medium (BD Bacto^™^, Sparks, MD, USA) at 37 °C for 20 h. Twenty-four healthy broiler chicks (One-day-old) were randomly assigned to the uninfected sham control (NV) and *C. perfringens* infection (CP) groups, with twelve birds per group. An antibiotic-free starter diet with 20% protein (BARC Feed Mill, Beltsville, MD, USA) was provided to the birds from days 1 to 14 post-hatch. From the 15th-day, a high-protein diet consisting of 10% protein diet and 50% fish meal was used to feed the birds. On day 20, the chickens were orally infected with 2.0 mL of *C. perfringens* culture (2.0~4.0 × 10^9^ colony-forming units per chicken), twice daily for five consecutive days, while uninfected sham control birds were orally given an equivalent volume of BYC culture medium. All the birds were weighed and sacrificed on day 5 post-first infection, and the sections (around 2.5 cm) of mid jejunum (with intestinal contents) were collected and instantly soaked in RNALater solutions (Sigma-Aldrich, St. Louis, MO, USA). The homogenized lysates of jejunum tissues with a Polytron homogenizer were incubated with 100 µL of Phenol:Chloroform:Isoamyl Alcohol (25:24:1, pH 6.8–8.0) for 15 min on ice after vigorous vortexing. A RNeasy PowerMicrobiome Kit (Qiagen Inc., Gaithersburg, MD, USA) was then used to extract RNA according to the manufacturer’s instructions. Freshly isolated RNAs from three randomly selected pooled samples in each group were sent to Genomic Resource Center (GRC), University of Maryland (Baltimore, MD, USA) for RNA sequencing. A 6-point scoring system was used to define gross pathological lesions in the intestines [26].

### 2.3. Library Construction and Sequencing Analysis

All RNA-sequencing services, including RNA quality determination, ribosomal RNA (rRNA) removal, library construction, and sequencing in HiSeq 2500 machine, were performed by GRC in a standard protocol [27]. Briefly, prior to library construction, an Illumina Ribo-Zero™ Plus rRNA Depletion Kit (Illumina, San Diego, CA, USA) was used to lower the level of rRNA present in total RNA samples. For sequencing on all Illumina platforms, the NEBNext^®^ Ultra™ II RNA Library Prep Kit for Illumina^®^ (New England Biolabs, Ipswich, MA, USA) was used to generate strand-specific libraries. The AMPure SPRI-select beads (Beckman Coulter Genomics, Danvers, MA, USA) were applied for the size selection of the library. Glycosylase digestion of the adapter and 2nd strand synthesis was carried out. Multiplexed libraries were sequenced on an Illumina NovaSeq 6000 S1 flowcell using a 101bp paired-end run. Data analysis was performed by Novogene Inc (Chula Vista, CA, USA). The software GOseq, topGO, and *hmmscan* release 2.12 were used to process whole-transcriptome sequencing data and implement differential gene expression (DGE) analysis, Gene Ontology (GO), and GO enrichment analysis as similarly described elsewhere [27]. GO annotation of upregulated or downregulated genes was carried out for functional classification based on the categories of biological processes, molecular function, and cellular components. The paired-end reads were aligned to the chicken genome (Galgal GRCg6a).

### 2.4. Statistical Analysis

The growth rate was calculated in the relative body weight gain percentage (RBWG%). GraphPad Premier 8.0 software (GraphPad Prism, San Diego, CA, USA) was used to perform the statistical analysis of the RBWG%, lesion scores, and transcriptional read counts of 5 major foodborne pathogens to total gut microbiota reads in small intestines by unpaired Student’s T-test. The identification of DEGs between the two different conditions was carried out using DEGseq software (v.1.10.1). The statistical significance for DEGs was set at the 0.05 significance level.

## 3. Results

### 3.1. Growth Underperformance Post C. perfringens Infection

**Figure 1:**
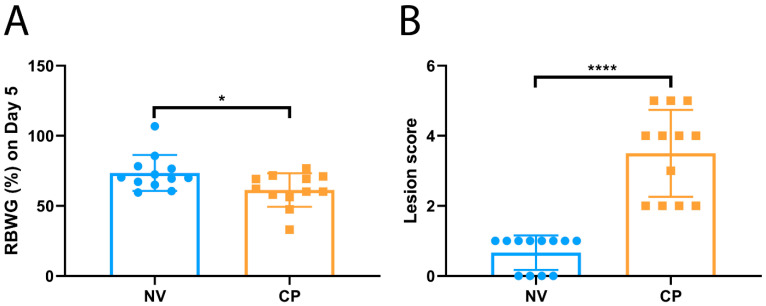
The impacts of *C. perfringens* infection on bird performance. The chicks were fed a high-protein diet. On day 20, the chickens were orally infected with *C. perfringens* culture (2.0~4.0 × 10^9^ colony-forming units per chicken), twice a day for five consecutive days. In addition, uninfected sham control birds were orally given an equivalent volume of BYC culture medium. All the birds were weighed and sacrificed on day 5 post-first infection. (**A**): The relative body weight gain (RBWG%) between sham control (NV, *n* = 12) and *C. perfringens*-infected groups (CP, *n* = 12). All birds were weighed before *C. perfringens* infection (CP0) and on day 5 post-infection (CP5). The following equation: (BW_CP5_–BW_CP0_)/BW_CP0_ × 100% was used to calculate the RBWG%. RBWG% values are represented as minimum to maximum (all points). (**B**): Lesion scores in sham control and *C. perfringens*-infected groups. Gross pathological lesions were evaluated using the jejunum sections. Gut lesion scores are shown as minimum to maximum (all points). Asterisks indicate significant differences between groups (* *p* < 0.05 and **** *p* < 0.0001).

Given that *C. perfringens*-induced NE results in the reduction in feed intake and lower growth rate of infected broiler chickens, the pattern of reduced body weight gain of inoculated chickens was expected in this study. When chickens were infected with pathogenic *C. perfringens*, a significantly lower growth rate in RBWG% was observed compared to the uninfected sham controls (61.3 ± 12.0% for CP vs. 73.5 ± 12.8% for NV, *p* < 0.05) (Figure 1A). In addition to the body weight gain, the growth performance of the infected and uninfected birds was also evaluated by gut lesion scoring. The inoculation of the *C. perfringens* TpeL17 strain significantly induced NE lesions, as shown by much higher gut lesion scores in the infected group when compared with the uninfected sham control group (0.67 ± 0.49 for CP vs. 3.5 ± 1.2 for NV, *p* < 0.0001) (Figure 1B).

### 3.2. Identification of DEGs in the Small Intestines between C. perfringens-Infected and Control Groups

**Figure 2:**
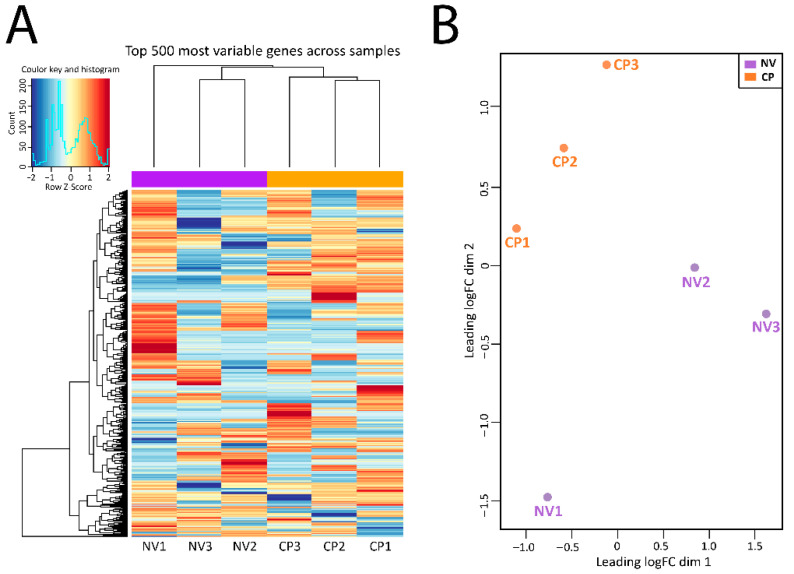
Mapping and analysis of RNA-seq data of chicken jejunum samples between sham control (NV: NV1, NV2, and NV3) and *C. perfringens*-infected groups (CP: CP1, CP2, and CP3). (**A**). Hierarchical clustering of 500 most variable genes across all samples in the heat map format. Log2 values for gene expression quantification were employed to cluster all these genes by hierarchical clustering. (**B**). Summary of the datasets between the sham control and *C. perfringens*-infected groups by multidimensional scaling (MDS) plots. The relationships between all pairs of samples were revealed by edgeR’s plotMDS using a count-specific distance measure.

The DGE analysis was carried out in the following steps as described elsewhere [27]: read count normalization, *p*-value estimation by negative binomial distribution model, false discovery rate (FDR) value estimation based on multiple hypothesis testing, and evaluation of gene expression levels with fragments per kilobase of transcript per million base pairs sequenced. The top 500 most variable genes across samples were graphically illustrated in the heat map (Figure 2A). The sample distribution of each group was inspected by the function plotMDSown and was shown in Figure 2B using multidimensional scaling plots. Generally, distinct separation contours exist between the CP infection group and the NV control group.

### 3.3. Identification of Highly Abundant Up-/Down-Regulated Genes in Response to C. perfringens Infection

To identify differentially up-/down-regulated host transcripts induced by *C. perfringens* infection, RNA-seq mapping was applied to yield quantitative DEG profiles. In this study, *C. perfringens*-infection triggered a series of events in transcription levels in the host small intestines in the chicken. In total, 13,473 annotated chicken genes showed differential expression in the two groups. RNA-Seq analysis of small intestinal samples in both groups unveiled 606 upregulated and 624 down-regulated genes (|fold change| > 1.5, *p* ≤ 0.05), among which 48 upregulated genes and 48 down-regulated genes were shown between these two groups (|fold change| ≥ 2.0, adjusted *p*-value FDR ≤ 0.05) at day 5 post *C. perfringens* infection in chicken jejunum (Table 1 and Table 2).

The upregulated genes with more than 2.0 folds (×) increase in the jejunum epithelial layer are summarized in Table 1. Among enriched up-regulated genes, top 10 transcripts with known functions are phosphocholine cytidylyltransferase 1 alpha (CCTα; 26.3 fold changes ×), chromobox 3 (CBX3, 25.7×), filamin B (FLNB, 25.3×), late endosome and lysosome associated marker (VPS33B, 24.2×), complex locus (SPEG, 20.5×), striatin (STRN, 18.1×), disheveled associated activator of morphogenesis (DAAM2, 16.4×), transcription factor 3 (TCF3, 12.9×), NACC family member (NACC2, 12.8×), and copper chaperone for superoxide dismutase (CCS, 12.6×) (Table 1). Intriguingly, the IL-17D gene related to signal transduction, the regulation of cytokine production, and inflammatory responses was also significantly enriched (4.1X) in *C. perfringens*-inoculated broiler chickens (Table 1), indicating the regulatory roles of IL-17 in *C. perfringens*-caused tissue damage and inflammation.

Differentially down-regulated genes with more than 2.0-folds decreases were also identified between these two groups (|fold change|≥ 2.0, adjusted *p*-value FDR ≤ 0.05) at 5th day post *C. perfringens* inoculation in bird jejunum layer (Table 2). Of the upmost 10 down-regulated transcripts with known functions, these genes encoded coronin 1C (CORO1C, −32.9X), protein tyrosine phosphatase receptor type (PTPRF, −29.5X), prosaposin (PSAP, −27.7X), eukaryotic translation initiation factor 4 (EIF4G1, −24.2X), microsemino protein beta (MSMB, −23.0X), ribosomal protein L7 (RPL7, −15.2X), tubulin folding cofactor (TBCD, −12.7X), eukaryotic translation initiation (EIF5, −12.5X), small ArfGAP 1 (SMAP1, −12.1X), and DENN domain containing 5B (DENND5B, −10.9X) (Table 2). Of note, a cascade of down-regulated genes, e.g., 15-hydroxyprostaglandin dehydrogenase (HPGD, −3.23X), retinol-binding protein-2 (RBP2, −2.90X), and fatty acid-binding protein 2 (FABP2, −2.44X), are associated with energy metabolism, suggesting energy imbalance in the pathogenesis of weight loss caused by pathogenic *C. perfringens* inoculation (Table 2). The gene encoding MHC Class I antigen (BF1) related to immunity was also down-regulated (−2.47X) (Table 2).

### 3.4. GO Annotation for DEGs

**Figure 3:**
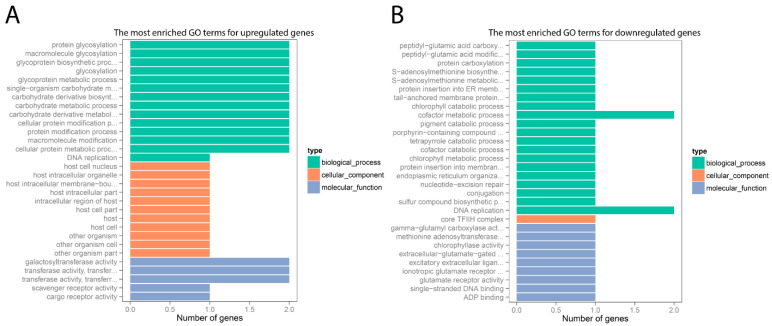
Functional analysis of upregulated (**A**) and downregulated (**B**) genes in chicken jejunum. The *x*-axis shows differentially expressed gene (DEG) numbers. The *y*-axis reveals the most enriched GO terms for DEGs. The GO terms of molecular function, cellular component, and biological process classes are compared based on numbers of upregulated and downregulated genes between *C. perfringens*-infected (CP) and sham control (NV) groups.

GO term enrichment analysis was used to determine biological categories in which DEGs were engaged. Figure 3 showed that the DEGs identified from *C. perfringens*-infected group were assigned to three main classes: molecular function, cellular components, and biological process. Of all the upregulated genes, 27 were annotated into 14 subcategories of biological process terms, which were mainly associated with protein modification and energy metabolism (Figure 3A). Besides, 11 upregulated genes were assigned to 11 subcategories of cellular components terms, and 8 were enriched in 5 subcategories of molecular function terms (Figure 3A). In addition, the downregulated genes in *C. perfringens*-infected group were allocated to 20 terms of the biological process class, 1 term of the cellular component class, and 9 of the molecular function class (Figure 3B).

### 3.5. The Mapping of C. perfringens and Other Foodborne Pathogens

**Figure 4:**
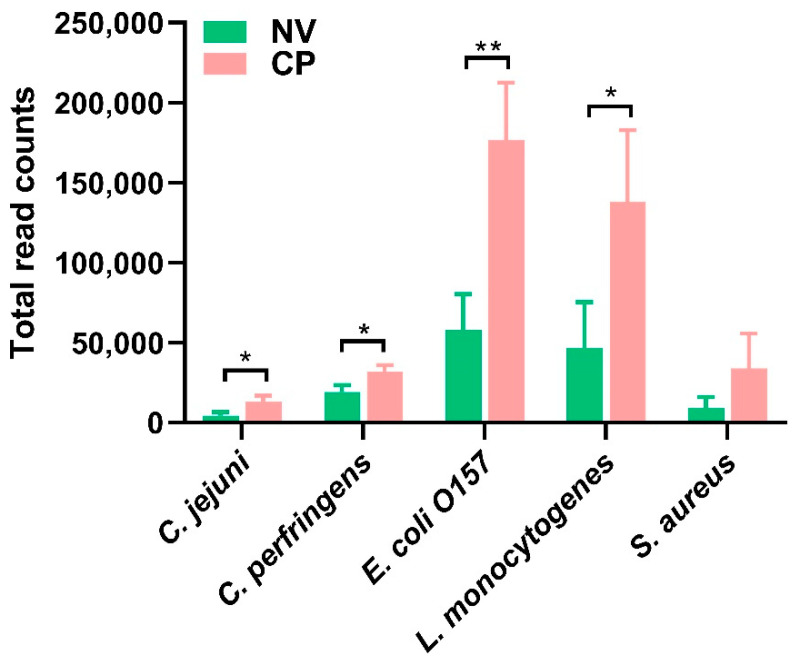
The relative ratios (*10^3^) of transcriptional read counts of five major foodborne pathogens to total counts in small intestines at Day 5 post *C. perfringens* infections (twice a day for five days by oral gavage). The RNA was extracted from contents, and mRNA was sequenced by RNA-seq. Statistical analysis for each bacterium in *C. perfringens* (CP) infected group was carried out using unpaired T-test. * means *p* < 0.05 and ** mean *p* < 0.01 when compared to the sham control (NV) group. These bacterial references used were *Listeria monocytogenes* (Gene Bank accession number CP019624.1), *Campylobacter jejuni* (AL111168.1)*, Staphylococcus aureus* (BX571856.1)*, and Escherichia coli* O157 (CP043539.1), and *C. perfringens* (BA000016.3).

Foodborne pathogens pose constant risks to human and animal health. In recent years, some enteric bacterial pathogens, such as *Campylobacter jejuni* and *Listeria monocytogenes,* have been among the top causes of human foodborne bacterial diseases linked to poultry and their products. Compared with the sham control, *C. perfringens*-challenged group had significantly increased transcriptional levels for *Campylobacter jejuni* (*p* = 0.03), *Escherichia coli* O157 (*p* = 0.008), *C. perfringens* (*p =* 0.02), and *Listeria monocytogenes* (*p* = 0.041), as shown by the relative ratios of pathogenes’ transcriptional read counts to total read counts (Figure 4). However, no significant difference was shown at the transcriptomic level for *Staphylococcus aureus* between these two groups (*p* = 0.14) (Figure 4). It is implied that these foodborne pathogens probably utilize the host environment to outgrow the intestinal microbiota following *C. perfringens* infection.

## 4. Discussion

Understanding the host-bacteria interaction during disease pathogenesis is vital for the development of early intervention measures. The interaction between the chicken intestine and *C. perfringens* during infection could initiate a series of events leading to the transcriptomic alteration at the pathogen-host interface. Parreira et al. [28] performed a global transcriptomic analysis in birds infected by the *netB*-positive CP1 strain on ligated intestinal loops by washing away the commensal microbiota. They found that *C. perfringens* gene expression profiles in vivo resembled those of *C. perfringens* grown in nutritionally deprived conditions, indicating that pathogenic *C. perfringens* responds well under distinct environmental conditions [28]. The results found herein clearly indicated that *C. perfringens* infection significantly impacted the gene expressions of the transcriptome in the small intestinal mucosa. Forty-eight genes in the host were upregulated with significant differences following *C. perfringens* infection, including top enriched CCTα, Chromobox 3, and Filamin B. Inflammation and lipid signaling are intermingled modulators of homeostasis and immunity. Therefore, inflammatory signaling is able to significantly affect lipid metabolism in the multiple tissues in infection [29]. It is also noteworthy that the increased accessibility of phospholipids resulting from the epithelial cell membrane, including phosphatidylethanolamine and phosphatidylcholine, is associated with intestinal epithelial damage during inflammation [30]. CCT is a rate-limiting enzyme for lecithin synthesis [31]. CCTα, as one of the most important genes for phosphatidylcholine biosynthesis and in phospholipid metabolism [29], is reported to be required for antigen-specific germinal center-derived antibody-secreting cells and memory B cells [32]. In this study, CCTα was upregulated in *C. perfringens*-infected chicken samples. As suggested, upregulation of CCTα could enhance the production of memory B cells and plasma cells in the development of humoral immunity in reaction to *C. perfringens* invasion, since B cell-specific deletion of CCTα resulted in lowered IgG production and increased IgM secretion to initiate humoral immunes [32].

CBX3 belongs to the heterochromatin protein 1 (HP1) family encoded by the chromobox genes. The HP1 family members are evolutionarily conserved and may function in many cellular events, including telomere function, gene regulation, and DNA repair. CBX3 protein has been shown to promote optimal co-transcriptional RNA processing through the initiation of the splicing machinery [33]. Upregulation of CBX3 in chicken small intestines post *C. perfringens* infection could be related to the increased activities of a cascade of cellular processes, including DNA repairing. The detailed mechanism may warrant further investigation. Filamin B (FLNB) is a cytoplasmic protein that controls the cytoskeletal network [34], and mutations of FLNB gene may lead to skeletal disorders [35,36]. Striated muscle preferentially expressed protein kinase (SPEG) is required for myocyte cytoskeletal development, and mutations in SPEG gene are related with centronuclear myopathy [37]. In our study, at Day 5 post *C. perfringens* infection, FLNB and SPEG were significantly upregulated, suggesting the association with increased cytoskeletal network activities and myocyte repairing/development.

In this study, a late endosome and lysosome associated marker was also upregulated, possibly related to the late stage of some antigens/toxin internalization in vacuoles at Day 5 post *C. perfringens* infection. The internalized epsilon-toxin of *C. perfringens* was once reported to be moved from early endosomes to late endosomes and lysosomes maximized at 60 min in MDCK cells with confocal microscopy [38]. WD-40 repeat protein, Striatin, is one member of the calmodulin-binding protein subfamily and plays an essential role in sustaining epithelial adhesion, integrity, and barrier functions between neighboring cells [39]. The up-regulation of striatin after *C. perfringens* infection suggests that the host may increase processing activities to maintain the integrity of cell adhesion and reduce the negative impact caused by *C. perfringens* challenge. In addition, the upregulated *DAMM2* gene may be related to the Wnt signaling pathway and planar cell polarity pathway.

Following *C. perfringens* infection, some genes in the host intestinal mucosa were differentially down-regulated, for example, Coronin-1C, Receptor Protein-Tyrosine Phosphatases (RPTPs), and Prosaposin. Coronin-1C (Coronin-3) is a homotrimeric F-actin binding protein. Coronin-1C protein is implicated to participate in various cellular events, such as cell migration, gene regulation, apoptosis, signal transduction, cell cycle progression, tumor cell metastasis, and the regulation of leukocyte-specific signaling events in innate immunity [40,41,42]. RPTPs are one of the superfamily of protein-tyrosine phosphatases and play an essential role in signal transduction across cell membranes. Thus, RPTPs are predominant drug targets in these various human pathologies [43,44]. Prosaposin (PSAP) gene encodes a glycoprotein that is a precursor for four cleavage products: saposins A, B, C, and D, which may facilitate the catabolism of glycosphingolipids for the hydrolysis in the lysosomal compartment [45]. Meanwhile, other down-regulated genes may be involved in various functions, including in the downregulation of overall protein synthesis rates in mammalian and yeast cells (eukaryotic translation initiation factor 4, eIF4G1) [46], affecting the mRNA expression levels of the nearby genes (microsemino protein beta) [47], regulating clathrin-dependent endocytosis (SMAP1) [48], programmed cell death 4 (PDCD4), multi signaling molecules that control multiple aspects of cardiovascular development (Semaphorins), and associated pathogenic mechanisms if mutated (calsyntenin 1: CLSTN1).

In this study, the transcriptomic read counts for several critical foodborne pathogens, such as *E coli O157*, *L. monocytogenes*, and *C. jejuni*, were also found to be increased following *C. perfringens* infection in the small intestine tissues. In a normal healthy situation, microorganisms in gut microbiota have co-developed with the host in a symbiotic relationship to maintain colonization resistance by metabolic benefits, promotion of immune homeostasis, and immune protection against pathogen colonization [30]. Therefore, we speculated that intestinal inflammation might be started by a specific pathobiontic bacterium, or a group of pathobionts normally present at low abundance in the intestine that may outgrow under favorable conditions, such as mutations in antimicrobial genes or other byproducts secreted by *C. perfringens*. Introduction of pathogenic *C. perfringens* may result in a lowered overall microbial diversity with a reduction of beneficial symbionts and an increase of pathobionts [30]. Besides, over-expanded host immune responses may ultimately result in inflammation that thereafter interrupts the intestinal homeostasis to favor a more extreme dysbiosis by various means, involving the generation of antimicrobials, mucin consumption, nutritional alterations, and metal use [30]. For example, recent literature indicated a positive correlation between *C. perfringens* and *C. jejuni* in cecal contents in the chickens [49]. It was hypothesized that the hydrogen produced by *C. perfringens* might likely be required for *C. jejuni* ‘s Ni/Fe hydrogenase for growth, enhancing *C. jejuni* colonization [49,50]. It merits further investigations of the potential correlation and interaction between pathogenic *C. perfringens* and other foodborne pathogens in the host mucosal immune system, so as to timely control the occurrence of pathogenic microorganism overgrowth.

Since *C. perfringens* mainly infected the small intestine, our current study was focused on the field of the mid-section of the jejunum. Nevertheless, it demonstrated a comprehensive regulation of the complex responses to *C. perfringens* infection through our methodology. It would be interesting to further investigate the detailed host-pathogen interaction based on the findings from our current data.

## 5. Conclusions

A comprehensive overview of the transcriptomic profiling was obtained to study the interaction between host and *C. perfringens* pathogen in the chicken NE model. Through comparative analysis of DEGs between uninfected and *C. perfringens*-infected jejunum samples, we identified many gene transcriptional alterations of the host in inflammation, adaptive immunity, muscle skeletal development, cellular process, metabolism, and energy production. In addition, it appeared that the transcriptional levels for three important foodborne pathogens were also significantly elevated in a synergic relationship with *C. perfringens* infection. In general, the results may enhance our understanding of the molecular mechanisms of host-*C. perfringens* interactions in chicken NE.

## Figures and Tables

**Table 1:** List of significantly upregulated genes in chicken small intestinal mucosa in response to *C. perfringens* infection.

Gene ID#	FDR	Chromosome	Gene Symbol	Description	Absolute Fold Change
ENSGALT00000099249	0.001	9	PCYT1A	phosphate cytidylyltransferase 1, choline, alpha	26.26
ENSGALT00000065275	0	2	CBX3	chromobox 3 (CBX3)	25.65
ENSGALT00000102383	0.026	12	FLNB	filamin B (FLNB)	25.32
ENSGALT00000080045	0	10	VPS33B,	late endosome and lysosome associated	24.22
ENSGALT00000047160	0.012	7	SPEG	complex locus [SourceNCBIgene; Acc429033] 0	20.46
ENSGALT00000017218	0.013	3	STRN	striatin [SourceHGNC Symbol;AccHGNC11424] 00	18.08
ENSGALT00000103665	0.044	31		NA	16.51
ENSGALT00000065256	0.025	3	DAAM2	dishevelled associated activator of morphogenesis	16.38
ENSGALT00000101515	0.008	28	TCF3	transcription factor 3	12.86
ENSGALT00000108349	0	17	NACC2	NACC family member	12.76
ENSGALT00000095627	0.005	33	CCS	copper chaperone for superoxide dismutase	12.64
ENSGALT00000082640	0.013	33	NACA	nascent polypeptide associated complex subunit	12.29
ENSGALT00000047943	0.001	15	DEP	domain containing 5 [SourceNCBI gene; Acc416956]	11.39
ENSGALT00000087703	0	1		NA	11.32
ENSGALT00000094693	0.001	26		NA	10.12
ENSGALT00000026339	0.039	3	GSTA2	Glutathione S-Transferase 2	9.41
ENSGALT00000012083	0.009	9	PCYT1A	phosphate cytidylyltransferase 1, choline, alpha	8.66
ENSGALT00000090554	0.018			NA	7.86
ENSGALT00000074687	0	20	MYL9	myosin, light chain 9, regulatory	7.79
ENSGALT00000014587	0.003	3	EPCAM	epithelial cell adhesion	6.86
ENSGALT00000091530	0.012	6		NA	6.45
ENSGALT00000095666	0	2	SLC4A7	solute carrier family 4 member	6.36
ENSGALT00000018245	0.024	4		NA	6.08
ENSGALT00000050930	0.013	23	PUM1	pumilio RNA binding	6.01
ENSGALT00000081307	0.001	2	STT3B	STT3B, catalytic subunit	5.98
ENSGALT00000067100	0.011	1	ATP2B1	ATese plasma membrane	5.52
ENSGALT00000063811	0.008	Z	ribosomal	ribosomal protein S23	5.36
ENSGALT00000107263	0.039	1	FNDC3A	fibronectin type III	5.30
ENSGALT00000099291	0.029	28	GATAD2A	GATA zinc finger domain containing	5.13
ENSGALT00000092961	0.019	25	MTMR11	myotubularin related protein 11 [SourceHGNC	4.46
ENSGALT00000092785	0.016			NA	4.29
ENSGALT00000060227	0.005	14	HBM	hemoglobin subunit mu	4.26
ENSGALT00000056811	0.012	30	DNM2	dynamin 2 [SourceHGNC Symbol;AccHGNC2974] 0	4.17
ENSGALT00000057600	0.013	1	IL17D	interleukin 17D [SourceHGNC Symbol;AccHGNC5984] 0	4.07
ENSGALT00000070149	0.045	31	DHDH	dihydrodiol dehydrogena	4.04
ENSGALT00000094063	0.045	16		NA	3.82
ENSGALT00000026767	0.026	3	PBK	PDZ binding kinase	3.82
ENSGALT00000033333	0.012	8	LMO4	LIM domain only	3.81
ENSGALT00000098491	0.004	6		NA	3.50
ENSGALT00000053882	0.031	17		NA	3.36
ENSGALT00000089781	0.001	1	DCN	decorin (DCN), mRNA	3.31
ENSGALT00000036373	0.04	1	HBBA	hemoglobin beta, subunit A [SourceNCBI	3.20
ENSGALT00000038226	0.035	15	SMTN	smoothelin [SourceNCBI gene; Acc100858894] 00	3.20
ENSGALT00000104581	0.016	2			2.66
ENSGALT00000043405	0.047	28	TIMM13	translocase of inner mitochondrial membrane	2.59
ENSGALT00000107499	0.047	18	SRSF2	serine/arginine-rich splicing factor	2.55
ENSGALT00000068151	0.023	MT	ND6	NADH dehydrogenase subunit 6 [SourceNCBI	2.28
ENSGALT00000045265	0.045	3	Cystatin	SourceUniProtKB/Swiss-Prot; AccP01038]0000	2.11

**Table 2:** List of significantly downregulated genes in chicken small intestinal mucosa in response to *C. perfringens* infection.

Gene ID#	FDR	Chromosome	Gene Symbol	Description	Absolute Fold Change
ENSGALT00000096799	0.045	#N/A		#N/A	−45.54
ENSGALT00000087336	0.021	15	CORO1C	coronin 1C (CORO1C)	−32.92
ENSGALT00000077706	0.002	8	PTPRF	protein tyrosine phosphatase, receptor type	−29.51
ENSGALT00000081103	0	6	PSAP	prosaposin	−27.67
ENSGALT00000049779	0	9	EIF4G1	eukaryotic translation initiation factor 4	−24.18
ENSGALT00000087331	0.016	6		microsemino protein beta	−22.96
ENSGALT00000078878	0.043	#N/A		#N/A	−22.35
ENSGALT00000066511	0.023	2	RPL7	ribosomal protein L7	−15.15
ENSGALT00000064276	0.028	18	TBCD	tubulin folding cofactor	−12.68
ENSGALT00000018692	0	5	EIF5	eukaryotic translation initiation	−12.48
ENSGALT00000096298	0.036	3	SMAP1	small ArfGAP 1	−12.08
ENSGALT00000021113	0	1	DENND5B	DENN domain containing 5B	−10.93
ENSGALT00000036292	0.005	12	PRKCD	protein kinase C	−10.82
ENSGALT00000098137	0	3	SPTBN1	spectrinbeta non-erythrocytic	−9.17
ENSGALT00000101130	0	9	PCYT1A	phosphate cytidylyltransferase 1, choline, alpha	−8.61
ENSGALT00000047339	0.05	4	SMAD1	SMAD family member 1	−8.45
ENSGALT00000095523	0.007	4	FAT1	FAT atypical cadherin 1	−8.33
ENSGALT00000096470	0	2	SLC4A7	Solute carrier family4 member	−6.63
ENSGALT00000107225	0	24	APOA4	apolipoprotein A4(APOA4)	−6.43
ENSGALT00000055069	0.003	2	SEMA5A	semaphorin 5A	−6.34
ENSGALT00000007275	0.027	20	NCOA3	nuclear receptor coactivator 3	−6.32
ENSGALT00000090409	0.05	1	TMCC3	transmembrane and coiled-coil domain family	−5.76
ENSGALT00000107684	0.001	6	PDCD4	programmed cell death 4	−5.31
ENSGALT00000015332	0.026	3	BROX	BRO1 domain and CAAX motif	−4.73
ENSGALT00000086950	0.023	2	YWHAZ	tyrosine 3-monooxygenase/tryptophan 5-monooxygenase	−4.63
ENSGALT00000017711	0.045	7	ABCB11	ATP binding cassette subfamily B	−4.50
ENSGALT00000022319	0.003	3	GTF2H5	general transcription factor	−4.37
ENSGALT00000104993	0.009	8	LMO4	LIM domain only	−4.26
ENSGALT00000004104	0.027	21	CLSTN1	Cal syntenin 1 (CLSTN1)	−3.90
ENSGALT00000007773	0.002	15	ISCU	iron-sulfur cluster assembly enzyme	−3.78
ENSGALT00000061711	0.004	23	PTP4A2	protein tyrosine phosphatase type IVA,	−3.53
ENSGALT00000006691	0.012	8	SOAT1	sterol O-acyltransferase 1	−3.32
ENSGALT00000032201	0.025	4	HPGD	15-hydroxyprostaglandin dehydrogenase (HPGD)	−3.23
ENSGALT00000039749	0.001	1	CD36	CD36 molecule (CD36)	−3.15
ENSGALT00000025653	0	3	EEF1A1	eukaryotic translation elongation factor 1	−3.12
ENSGALT00000105502	0.017	#N/A		#N/A	−2.90
ENSGALT00000008547	0.001	9	RBP2	retinol binding protein	−2.90
ENSGALT00000066968	0	#N/A		#N/A	−2.83
ENSGALT00000063525	0.045	6	CYP2C18	cytochrome	−2.79
ENSGALT00000018393	0.025	2	RAB5A	RAB5A, member RAS	−2.74
ENSGALT00000024116	0.026	3	SOT3A1L	sulfotransferase family 3A	−2.56
ENSGALT00000052923	0.023	11	CA7	carbonic anhydrase 7	−2.54
ENSGALT00000089467	0.049	16	BF1	MHC BF1	−2.47
ENSGALT00000019554	0.043	4	FABP2	fatty acid binding protein 2	−2.45
ENSGALT00000083719	0.012	2	MEP1B	meprin A subunit beta	−2.39
ENSGALT00000086032	0.028	1	GAPDH	glyceraldehyde-3-phosphate dehydrogenase	−2.33
ENSGALT00000062517	0.035	#N/A		#N/A	−2.21
ENSGALT00000004055	0.028	15	RAN	RAN, member RAS	−2.15

## Data Availability

The datasets supporting the findings of this article are included within the article. The additional datasets will be available from the corresponding author upon reasonable request.

## Abbreviations

NE	necrotic enteritis
RNA-seq	RNA sequencing
NV	uninfected sham control
RBWG%	relative body weight gain percentage
GO	Gene Ontology
DGE	Differential gene expression
DEG	Differentially expressed genes
FDR	False discovery rate
